# Amelioration of oxygen-induced retinopathy in neonatal mice with fetal growth restriction

**DOI:** 10.3389/fcell.2024.1288212

**Published:** 2024-02-16

**Authors:** Ryusuke Watanabe, Shuang Liu, Tomohisa Sakaue, Yasuhito Ikegawa, Masaaki Ohta, Takashi Higaki, Masaki Mogi, Mariko Eguchi

**Affiliations:** ^1^ Department of Pharmacology, Ehime University Graduate School of Medicine, Matsuyama, Ehime, Japan; ^2^ Department of Pediatrics, Ehime University Graduate School of Medicine, Matsuyama, Ehime, Japan; ^3^ Department of Cardiovascular and Thoracic Surgery, Ehime University Graduate School of Medicine, Matsuyama, Ehime, Japan; ^4^ Department of Cell Growth and Tumor Regulation, Proteo-Science Center (PROS), Matsuyama, Ehime, Japan; ^5^ Department of Ophthalmology, Ehime University Graduate School of Medicine, Matsuyama, Ehime, Japan; ^6^ Department of Regional Pediatrics and Perinatology, Ehime University Graduate School of Medicine, Matsuyama, Ehime, Japan; ^7^ Department of Regional Child Health Care, Ehime University Graduate School of Medicine, Matsuyama, Ehime, Japan

**Keywords:** retinopathy of prematurity, neonate, fetal growth restriction, neovasculature, MAPK signaling pathway

## Abstract

**Introduction:** With the aim of optimizing the balance of maintaining a safe oxygen saturation and reducing the risk of retinopathy of prematurity in human neonates with fetal growth restriction (FGR), the present study investigated the distinct effects of oxygen supplementation on the retinal neovasculature using a murine premature neonatal oxygen-induced retinopathy (OIR) model with or without fetal growth restriction.

**Methods:** For comparison with normal birth-weight neonates, maternal low-protein diet-induced FGR neonates were subjected to fluctuating oxygen levels to generate oxygen-induced retinopathy. The retinal neovasculature was histologically evaluated, and comprehensive transcriptome analysis was conducted.

**Results:** Compared to OIR neonates with normal birth weight, significant amelioration of the neovasculature, as indicated by decreases in the number of branch junctions, vascular distribution, maximal vascular radius and microaneurysm-like tufts, was observed in OIR mice with FGR. The results of retinal RNA-sequencing revealed downregulation of angiogenic factors that trigger pathological retinal neovascularization, such as the mitogen-activated protein kinase pathway and corresponding upstream signaling pathways in OIR mice with FGR.

**Conclusion**: Our findings demonstrated that FGR neonates have a higher capacity for retinal oxygen stress, and the risk of OIR development is attenuated compared to that in mature neonates with normal birth weight.

## Introduction

Supplemental oxygen is effective and essential for initial resuscitation and neonatal intensive care. The initial delivered oxygen concentration strictly depends upon gestation, with 21% oxygen for ≥32 weeks, 21%–30% oxygen for 28–32 weeks, and 30% oxygen for <28 weeks of gestation (Madar et al., 2021). For infants’ intensive care, precise titration of oxygen delivery by measuring oxygenation in neonates is recommended. A target percutaneous oxygen saturation (SpO_2_) range of 85%–95%, which corresponds to an arterial partial pressure of oxygen (PaO_2_) range of 29–67 mmHg (3.8–8.9 kPa), in both mature and premature oxygen-dependent neonates in the first 2 weeks of life is recommended (Saugstad, 2018). The reason for the relatively strict limitation of the concentration of supplemental oxygen is that hyperoxia-related oxidative stress has been associated with a multitude of adverse outcomes (Saugstad, 2018). Among these adverse outcomes, retinopathy of prematurity (ROP), underdevelopment of the retina that results in aberrant growth of the retinal vasculature, is considered an early risk factor for neonatal blindness or visual impairment (Hartnett and Penn, 2012). According to a recent study conducted in preterm monochorionic twin pairs with a birth weight discordance ≥20%, FGR is critically associated with the development of ROP in premature infants. The incidence of ROP was higher in the twin with FGR compared to the twin without FGR, 30% (9/30) *versus* 12% (4/30), respectively (OR 2.8, 95% Cl: 1.2–6.6) (Spekman et al., 2023).

The default understanding of most neonatologists is that preterm/premature newborns, especially those with fetal growth restriction (FGR), may be more sensitive to oxidant injury compared to normal newborns because of immaturity and decreased oxidant protective mechanisms, such as antioxidant enzymes and vitamins (Lee and Chou, 2005). Indeed, it has been reported that the incidence of ROP is particularly high (48–62%) in infants with a gestational age of 30 weeks or less, or with an extremely low birth weight of 1,500 g or less (Painter et al., 2015; Reyes et al., 2017; Fierson, 2018). However, with a focus on ROP in FGR neonates, the evidence for using less, or indeed more, oxygen in neonatal intensive care continues to be unclear. Therefore, it is useful to ask the question, what is the sensitivity of the premature retina to oxygen stress? To avoid ROP, should we pay more attention to the quantitative limitation of supplemental oxygen in premature infants, who often require extra oxygen for breathing support compared to mature infants because of both decreased antioxidant defenses and pulmonary immaturity?

To answer these questions, assessment of the development of ROP was performed using an established murine OIR model of maternal low-protein (LP) diet-induced FGR neonates with low birth weight (Chisaka et al., 2016). The OIR model is a well-established model which has been widely used to study signaling pathways involved in ROP. Newborn pups are exposed to a hyperoxic environment and develop avascular retinas from vaso-obliteration of newly formed capillaries in the central retina (Wang, 2016). After pups are moved to room air, the avascular central retina becomes hypoxic and intravitreal neovascular tufts are formed at the junction of the vascular and avascular retina. Mature pups that are placed in a hyperoxic environment already have complete primary plexus vascularization of the retina, which is different from preterm infants whose retinas are incompletely vascularized. Therefore, this OIR has been considered to not precisely mimic the pathological conditions seen in human ROP. By establishing a systemic FGR status along with a decrease in angiogenesis in an OIR model (Chisaka et al., 2016), in which delayed physiological retinal vascular development is to be expected, our FGR-OIR model could be a more representative model of human ROP.

In an effort to elucidate the vulnerability of the premature retina to oxygen-induced stress and contribute valuable insights into optimal oxygenation strategies for neonatal intensive care to mitigate ROP, we conducted a comparative investigation involving neonates from dams exposed to a normal-protein (NP) diet. Neonates with FGR were exposed to fluctuating oxygen levels to induce OIR, and subsequent assessment of retinal neovascularization was performed. Given that infants with FGR exhibit heightened susceptibility to OIR owing to compromised antioxidant defenses, pulmonary immaturity, and diminished retinal angiogenesis, our hypothesis posits that exposing neonates with FGR to fluctuating oxygen levels will result in a greater incidence of OIR compared to neonates with normal growth under similar oxygen conditions.

## Materials and methods

### Animals and diets

All animal experiment protocols were performed in accordance with the National Institutes of Health guide for the care and use of laboratory animals and approved by the Ehime University Committee for Animal Research (approval no. 05KI42-1).

Eight-week-old C57BL/6J female and male mice were obtained from CLEA Japan (Tokyo, Japan). A murine premature neonatal model was induced by maternal protein restriction, as we described previously (Chisaka et al., 2016). Female C57BL/6J mice were fed a 23% NP diet or 8% LP diet (Oriental Yeast, Tokyo, Japan) from 10 weeks of age until delivery. Male mice were fed NP diet, and mating was conducted at 12 weeks of age. After the day of delivery, all dams were returned to NP diet.

### Induction of OIR in neonates

A neonatal OIR model was established using a two-stage induction protocol as previously described (Scott and Fruttiger, 2010; Matsuda et al., 2017). Briefly, offspring (15 pups/group) and their nursing mothers were housed in an exposure chamber. Animals were subjected to oxygen loading (75% O_2_ and 25% N_2_ by volume) or mixed air loading (21% O_2_ and 79% N_2_ by volume), as a negative control, from postnatal day (P) 7 to P12 (Kiyoi et al., 2020). Mice were then placed in room air for an additional 5 days (P12-17). On P17, the offspring were anesthetized and the contralateral retinas were dissected for subsequent angiogenic evaluation and transcriptome analysis.

### Evaluation of retinal vasculature

Eyes were removed from offspring and pre-fixed for 1 h in 4% paraformaldehyde for whole-mount analysis. Retinas without retinal pigment epithelium were dissected and incubated overnight at 4°C with Alexa Fluor 488-conjugated isolectin B4 (IB4) from *Griffonia simplicifolia* (ThermoFisher Scientific, Waltham, MA). The retinas were then incubated with monoclonal anti-α-smooth muscle actin (SMA) antibody (1A4, ThermoFisher Scientific) for 2 h at room temperature, following by secondary labeling using Alexa Fluor 568-conjugated anti-mouse antibody (ThermoFisher Scientific). Subsequently, the retinal cups were flat-mounted for fluorescence observation. Images were captured using a fluorescence microscope BZ-X800 (Keyence, Osaka, Japan) or Nikon A1 confocal laser microscope (Nikon, Tokyo, Japan) and analyzed using ImageJ software and Angio Tool 0.6 (Informer Technologies, Inc.) according to established protocols (Zudaire et al., 2011).

### RNA extraction and transcriptome analysis

Total RNA was isolated from each retina and processed separately using a NucleoSpin RNA Plus XS kit (TaKaRa, Tokyo, Japan), and a library was subsequently prepared using a Switching Mechanism At the 5’ end of RNA Template (SMART) method. Purified amplicon was sequenced using a Novaseq 6000 sequencer (Illumina K.K. San Diego, CA). Raw bead counts were obtained and processed using a web portal for integrated differential expression and pathway analysis (iDEP.95/iDEP1.0; http://bioinformatics.sdstate.edu/, accessed on 24 February 2023) (Ge et al., 2018). k-Means clustering was performed on the top 2000 genes ranked by standard deviation into groups based on their expression pattern across all samples. A heatmap was subsequently generated in which numerical values of points were represented by a range of colors. Identification of differentially expressed genes (DEGs) was performed using a DESeq2 algorithm by extraction with an FDR cutoff of 0.1 and min-fold change of 2 as the default setting. Pathway analysis was performed using Parametric Gene Set Enrichment Analysis (PGSEA) with gene sets from the Gene Ontology Molecular Function and Pathway and the Kyoto Encyclopedia of Genes and Genomes (KEGG). The cutoff for significance was set at 0.2.

### Immunoblot analysis

Fresh retinas were isolated on P17 from NP_75% O_2_ and LP_75% O_2_ neonates and homogenized in RIPA buffer (ThermoFisher Scientific) with HALT protease and phosphatase inhibitors (ThermoFisher Scientific). The retinal extract was separated using a Jess system (ProteinSimple, San Jose, CA), using a 12–230 kD separation module and an anti-rabbit detection module, according to the manufacturer’s instructions. All primary antibodies, including angiogenesis antibody sampler kit (#8696: phospho-Akt, phosphor-Src, phosphor-PLCγ1, phosphor-p44/42 MAPK), HIF-1α (#36169), and GFAP (#80788) were obtained from Cell Signaling Technology (Cell Signaling Technology, Danvers, MA). The areas of target peaks were normalized by total protein within the same capillary using a RePlex feature and total protein assay (ProteinSimple). Data analysis was performed using Compass software for Simple Western ver.4.1.0 (ProteinSimple).

### Statistical analysis

All experiments were designed in a completely randomized multifactorial format. Sample distributions were analyzed using the Kolmogorov-Smirnov test. Mann-Whitney *U* test was used for nonparametric interclass comparisons with respect to birth weight. Differences among multiple groups were analyzed by two-way ANOVA, followed by Turkey test. *p* < 0.05 was considered significant. Results were expressed as mean ± standard deviation (SD). All data were analyzed using GraphPad Prism 9.5.1 (GraphPad Software, La Jolla, CA, United States).

## Results

### Oxygen stress caused OIR in NP neonates

**FIGURE 1:**
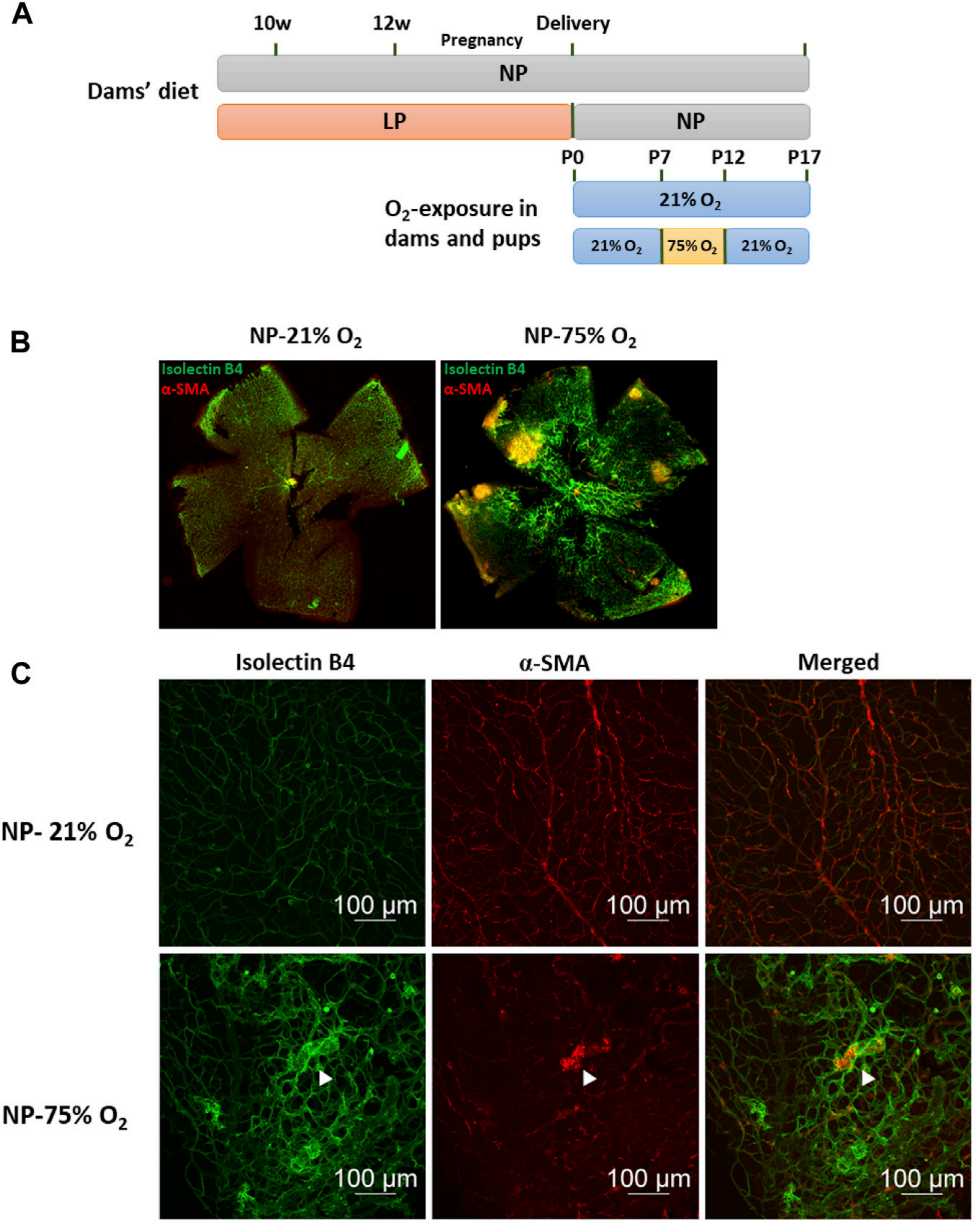
Oxygen-induced retinopathy in neonates with maternal normal-protein (NP, 20%) diet. **(A)** Establishment of maternal low-protein (LP, 8%) diet-induced neonatal FGR model and study design of OIR induction. Fifteen animals in each group were used for subsequent vasculature evaluation. **(B)** Representative images of flat-mounted retina in neonates with maternal normal-protein diet exposed to normoxia (NP-21% O_2_) or hyperoxia (NP-75% O_2_) on P17. The vasculature was labeled with isolecin B4 (green) and α-SMA (red). **(C)** Representative images of retinal vasculature in NP-21% O_2_ and NP-75% O_2_ neonates. NP, neonates from dams fed normal protein (20%); LP, neonates from dams fed low protein (8%) (white arrows: microaneurysm-like tufts).

Oxygen supplementation-induced retinopathy was induced using a two-stage O_2_-exposure protocol and used as a mouse ROP model (Figure 1A). A highly organized vascular plexus with a distribution of superficial and deep vascular networks reached the retinal periphery in NP neonatal mice on P17, as shown in representative images of whole-mounted retina (Figure 1B). Neonatal mice exposed to hyperoxia (75% O_2_) for 5 days (P7-P12) showed a temporal pattern of central retinal vascular insufficiency followed by neovascularization after a return to normal oxygen level (21% O_2_) for 5 days (P12-P17) (Supplementary Figure S1). P17 retina exhibited tortuous and dilated blood vessels (Figure 1C). Also, abnormal vascular structures such as tufts and ridges were observed. The hyperoxia retinal vasculature showed obvious morphological differences to the normal retinal vasculature, and an OIR model was thus successfully established.

### Vasculature in maternal low-protein-diet-induced FGR neonates

**FIGURE 2:**
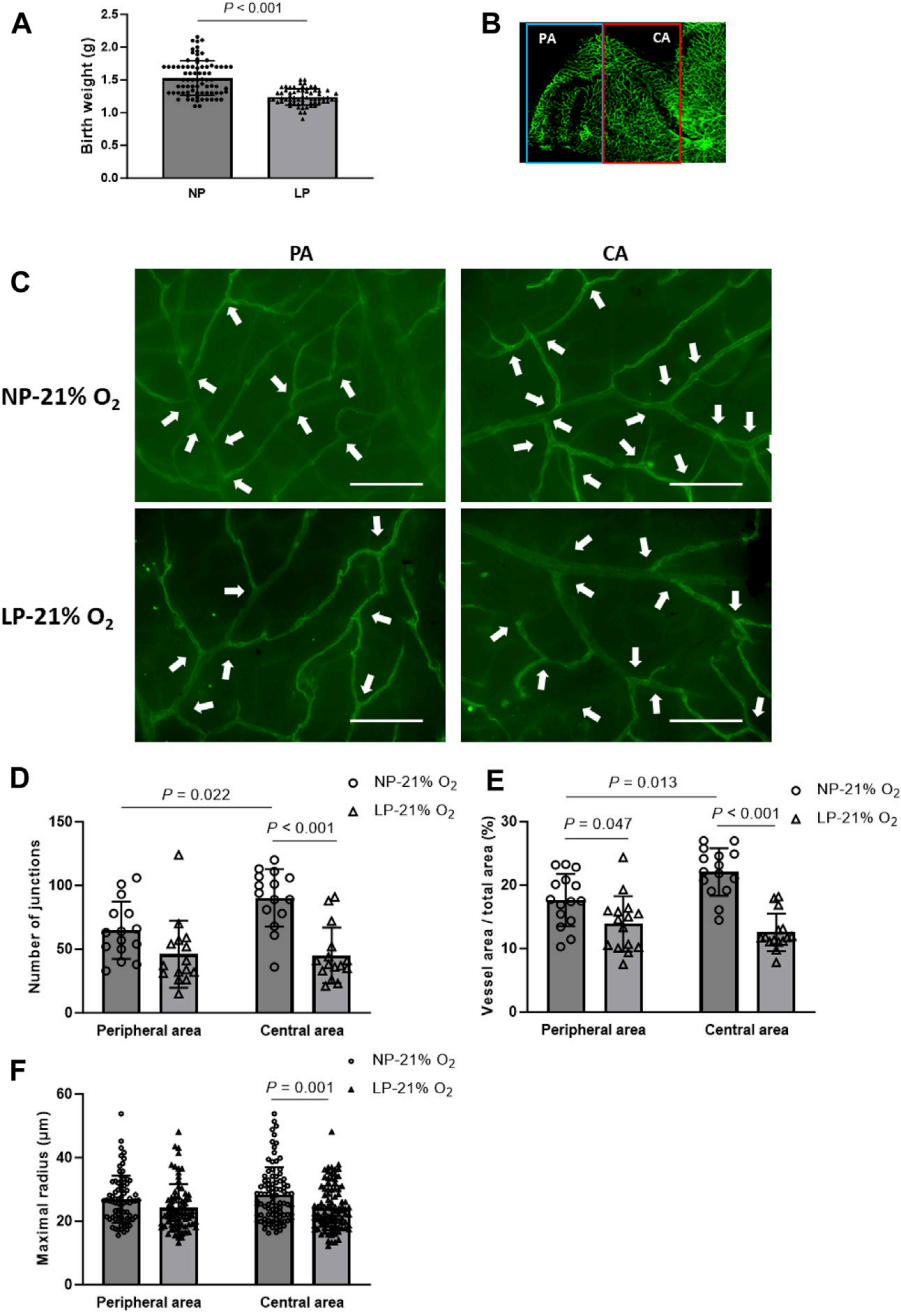
Vasculature in maternal low-protein (LP, 8%) diet-induced FGR neonates. **(A)** Birthweight of newborns from normal-protein (NP, 20%) diet and LP diet dams (n = 78 (NP) and n = 65 (LP)). Results are presented as mean ± SD. **(B)** Representative images of retinal vasculature in peripheral area (PA) and central area (CA). **(C)** Representative images of retinal vasculature in PA and CA in mature (NP-21% O_2_) and FGR (LP-21% O_2_) mice on P17. Arrowheads indicate branch junctions. Scale bar: 50 μm. **(D)** Number of vascular branch junctions, **(E)** relative area of vessel area, and **(F)** maximal radius were quantified in both PA and CA retinal vasculature of mature and FGR neonates. (n = 15; For maximal radius, 5-6 vessels in each retina were evaluated.) All results are presented as mean ± SD.

A maternal LP-diet caused a significant decrease in mean birth weight of newborns to 81.33% of that in maternal NP-diet newborns (1.50 ± 0.28 g vs. 1.22 ± 0.27 g) (Figure 2A). At P17 of normoxia, the hierarchical vascular network of the retinal cup was observed in both FGR neonates (LP-21% O_2_) and normal controls (NP-21% O_2_) (Figure 2B). The number of vascular branch junctions, the relative vascular area and the maximal radius of vascular branches were used to evaluate retinal vessel development. Compared to the peripheral area of the retina, vascular development in the central retina showed a mature developmental pattern in normal neonates, as indicated by increased values of branch junctions and vessel area (Figures 2C, D). A notably premature vascular developmental pattern was observed in LP-21% O_2_ neonates, particularly in the central retina, with a 49.93% decrease in branch junctions (45.2 ± 21.77 vs. 90.27 ± 22.56), a 43.03% decrease in relative vessel area (12.58% ± 2.93% vs. 22.08% ± 3.74%), and a 15.61% decrease in maximal vessel radius (24.12 ± 7.01 µm vs. 28.41 ± 8.63 µm), compared to NP-21% O_2_ neonates. Taken together, these findings indicate that maternal LP-induced FGR neonates showed impaired development of the retinal vasculature.

### Suppression of OIR in FGR neonates

**FIGURE 3:**
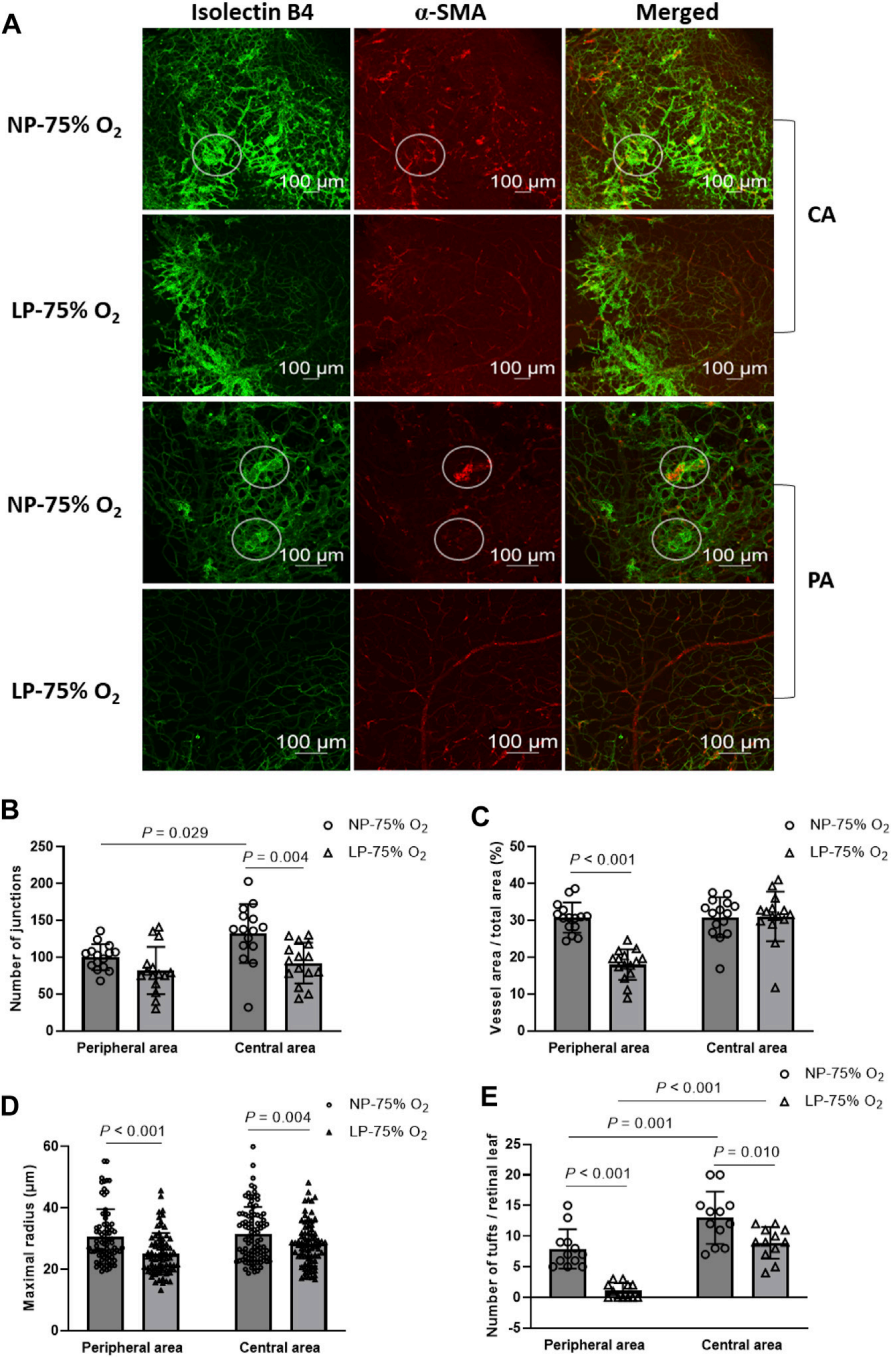
Amelioration of OIR in FGR neonates. **(A)** Representative images of both central and peripheral OIR retina in maternal normal-protein (NP, 20%) diet neonates exposed to hyperoxia (NP-75% O_2_) and maternal low-protein (LP, 8%) diet-induced FGR neonates exposed to hyperoxia (LP-75% O_2_). The vasculature was labeled with isolecin B4 (green) and α-SMA (red) and observed on P17. White circles: tuft formation. CA: central area; PA: peripheral area. **(B)** Number of vascular branch junctions, **(C)** relative vessel area, **(D)** maximal radius, and **(E)** number of microaneurysm-like tufts were quantified in both the peripheral and central retinal vasculature of mature and premature neonates. (n = 15; For maximal radius, 5-6 vessels in each retina were evaluated. For quantification of retinal tufts, the number of tufts in each retinal leaf and 4 leaves per retina were expressed.) All results are presented as mean ± SD.

OIR was established in both FGR and control neonates. Representative images of OIR retina are shown in Figure 3A. On P17, a significant decrease in the number of branch junctions was observed in the central retina, while the vascular distribution, as indicated by relative vessel area, was suppressed in the peripheral retina in FGR-OIR neonates compared to those in control OIR neonates (Figures 3B–D). More narrow vessels with diminished maximal vascular radius and microaneurysm-like tufts were observed in both the central and peripheral area of the retina in FGR neonates compared to those in control neonates. These results suggest that neovascularization was ameliorated in FGR neonates. This could be due to lower sensitivity of the retina to fluctuating oxygen levels compared to that in control neonates.

### Downregulation of MAPK signaling pathway in OIR neonates with FGR

**FIGURE 4:**
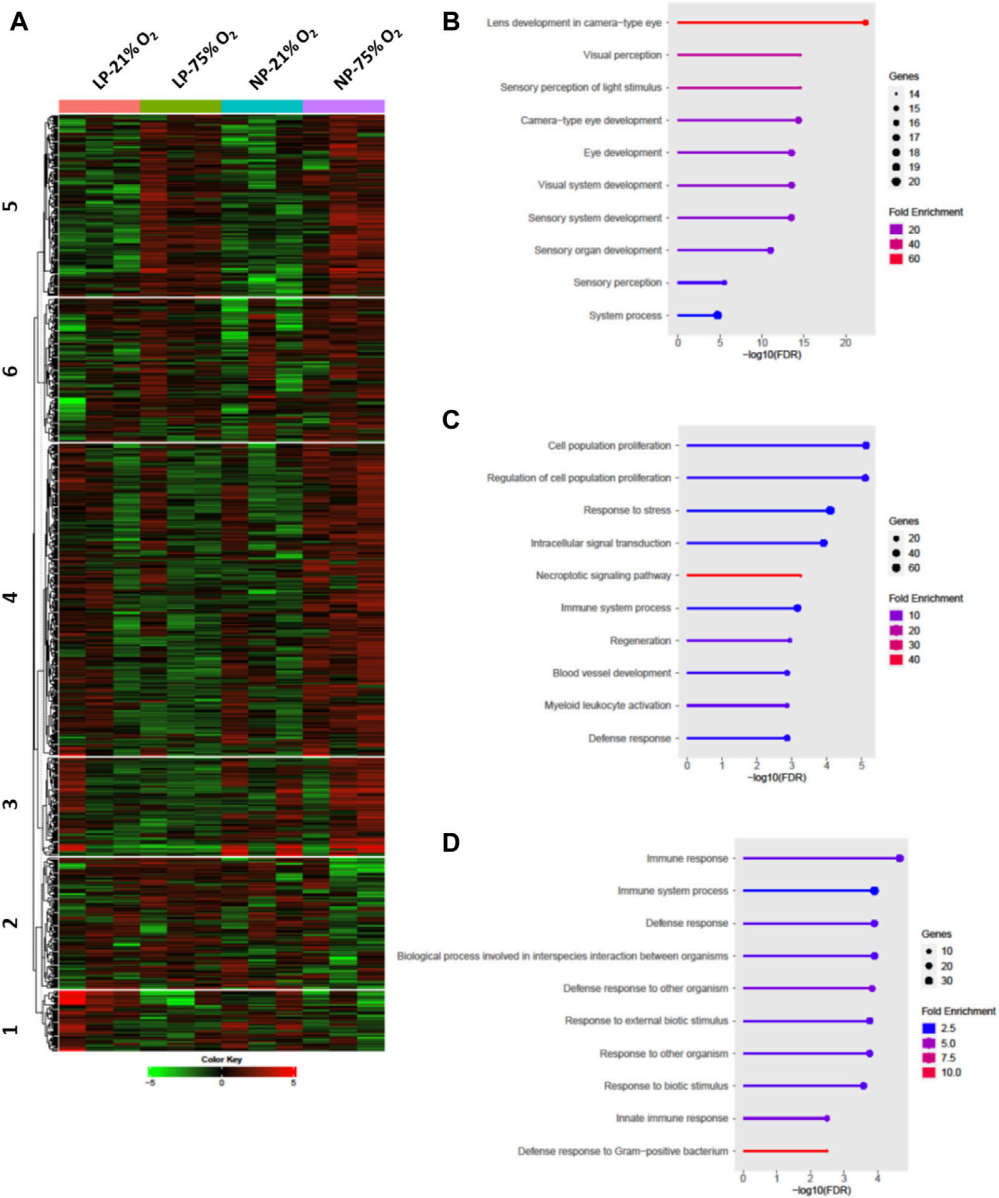
Transcriptome analysis in retinas from control and FGR neonates. **(A)** Heatmap and *k*-means clustering revealed 6 major clusters of the top 1,000 expression of genes in LP-21% O_2_, LP-75% O_2_, NP-21% O_2_, and NP-75% O_2_ neonatal retinas. Expression is displayed as log_2_ (fold-change). The results of Gene Ontology enrichment analysis, which revealed the genes included in **(B)** cluster 1, **(C)** cluster 4, and **(D)** cluster 6, are expressed using bar plots. NP, neonates from dams fed normal protein (20%); LP, neonates from dams fed low protein (8%).

Comprehensive transcriptome analysis was employed to elucidate the underlying mechanism of pathological changes in OIR neonates with FGR. Heatmap analysis of the top 1,000 upregulated and downregulated genes in the NP-21% O_2_, NP-75% O_2_, LP-21% O_2_, and LP-75% O_2_ groups indicated alteration of the transcriptome landscape in FGR retinas (Figure 4A and Supplementary Materials S2). These genes functionally belonged to six clusters. Enrichment analysis revealed that the genes included in cluster 1 were initially related to the visual sensory system, while the genes in cluster 4 were associated with cell proliferation and vascular development (Figures 4B, C). Interestingly, an immune-related gene cluster, cluster 6, was also identified in the FGR retinal transcriptome landscape (Figure 4D).

**FIGURE 5:**
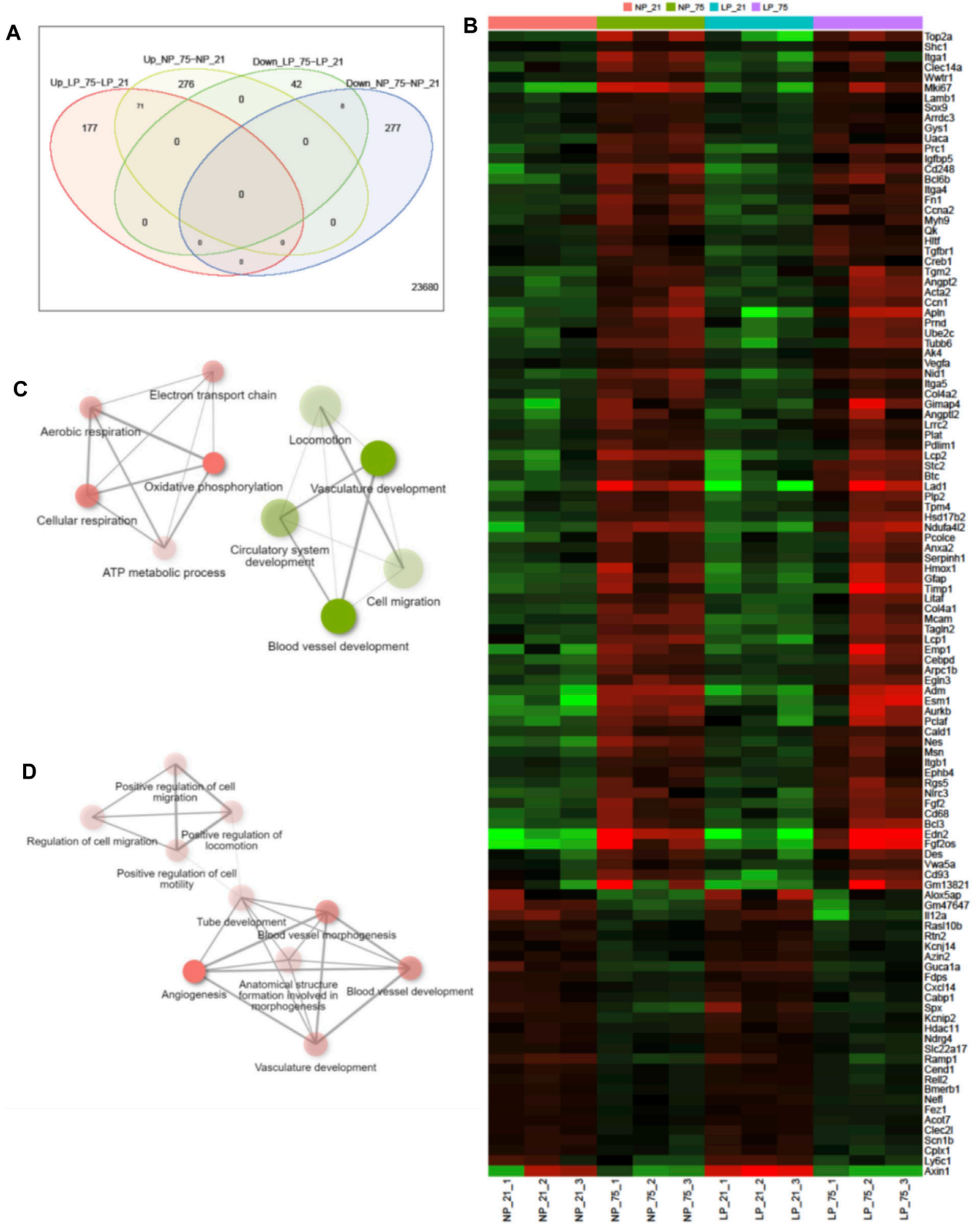
Alteration of gene expression in control and FGR OIR neonates. **(A)** Venn diagram showing number of upregulated and downregulated genes in comparisons of NP-75% O_2_ vs. NP-21% O_2_ and LP-75% O_2_ vs. LP-21% O_2_, identified by retinal RNA-seq. **(B)** Expression levels of DEGs shared in both NP-75% O_2_ vs. NP-21% O_2_ and LP-75% O_2_ vs. LP-21% O_2_ comparisons. Values are log_2_-transformed expression of DEGs. Network of the top 10 enriched pathways by Gene Ontology enrichment analysis in the comparison of **(C)** control neonates (NP-75% O_2_ vs. NP-21% O_2_) and **(D)** FGR neonates (LP-75% O_2_ vs. LP-21% O_2_) (cut off = 0.3). Red circle: upregulated pathway: green circle: downregulated pathway. NP, neonates from dams fed normal protein (20%); LP, neonates from dams fed low protein (8%).

Among the differentially expressed genes (DEGs), control neonates and FGR neonates shared 71 upregulated genes and 8 downregulated genes, which are assumed to be common factors related to oxygen-induced OIR in both control and FGR neonates (Figures 5A, B). In control OIR neonates, GO pathway enrichment analysis of DEGs revealed that mitochondrial functions, including electron transport chain, cellular respiration, oxidative phosphorylation, ATP metabolic process, and aerobic respiration pathways, were upregulated compared to those in maternal normal diet neonates without OIR (Figure 5C). Pathways related to vascular development, such as cell migration and locomotion, circulatory system development and blood vessel development were downregulated in control OIR neonates compared to mice without OIR. On the other hand, all 10 top enriched pathways in the comparison of FGR groups (OIR neonates vs. neonates without OIR) were upregulated. Pathways involved in cell motility, migration and locomotion were significantly upregulated, with a distinct in NP_75% O_2_ vs. NP_21% O_2_. Moreover, along with marked upregulation of angiogenesis, functional enhancement of blood vessel morphogenesis, tube development, and vasculature development was also predicted in FGR-OIR mice compared to FGR mice without OIR (Figure 5D).

**FIGURE 6:**
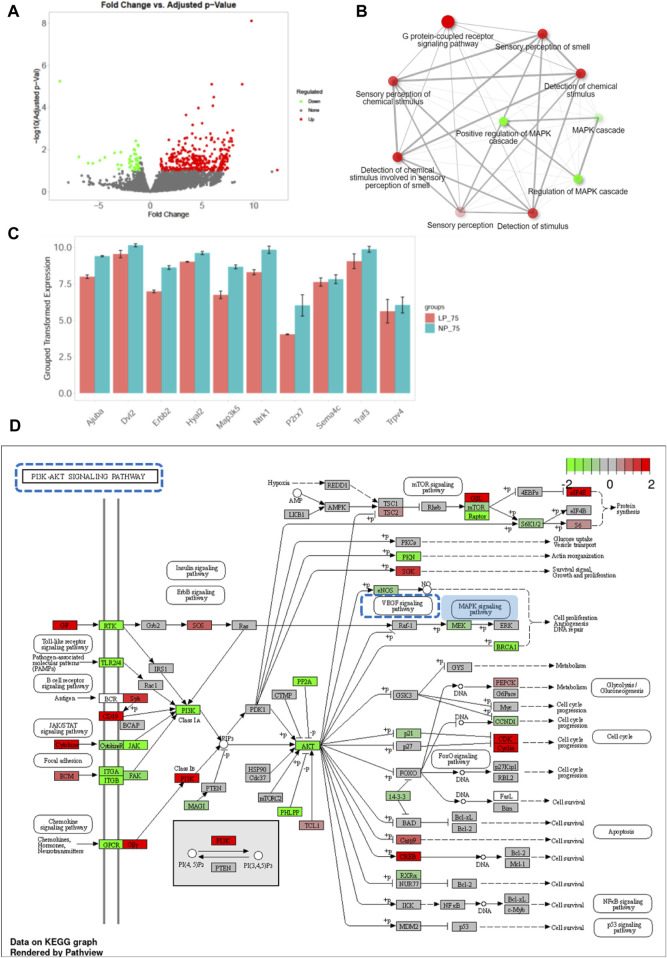
Downregulation of MAPK signaling pathway in OIR neonates with FGR. **(A)** Volcano plot of DEGs between control and FGR OIR neonates. Significantly downregulated genes are in green, upregulated genes are in red, and non-significant genes are in black. **(B)** Network of top 10 enriched pathways using Gene Ontology enrichment analysis in comparison of LP-75% O_2_ vs. NP-75% O_2_. Red circle: upregulated pathway: green circle: downregulated pathway. **(C)** Expression of key DEGs involved in MAPK signaling pathway. Results are presented as mean ± SEM. **(D)** KEGG pathway analysis was performed and the gene expression pattern of the PI3K-AKT signaling pathway is shown (LP-75% O_2_ vs. NP-75% O_2_). Red: upregulated genes; green: downregulated genes; square with blue dotted line: MAPK pathway-related signaling pathway. NP, neonates from dams fed normal-protein (20%); LP, neonates from dams fed low-protein (8%).

In the comparison between control OIR and FGR OIR neonates, 391 upregulated and 51 downregulated DEGs were detected (Figure 6A). GO pathway enrichment analysis of DEGs revealed that along with upregulation of the sensor-related pathway and G protein-coupled receptor signaling pathway, function of the mitogen-activated protein kinase (MAPK) cascade was downregulated in OIR neonates with FGR (Figure 6B). Expression of key genes related to the MAPK signaling pathway was significantly decreased in FGR OIR neonates compared to control OIR groups (Figure 6C). An altered gene expression map of the phosphatidylinositol-3 kinase-serine-threonine protein kinase (PI3K-AKT) signaling pathway, in which the genes upstream of the MAPK signaling pathway are annotated, is shown in Figure 6D. Certain signaling elements within the MAPK pathway/angiogenesis pathway, such as phospho-Akt, phospho-p44/42 MAPK, and phospho-Src, showed a trend of reduced protein levels in LP_75% O_2_ neonates compared to NP_75% O_2_ neonates, although this difference was not statistically significant (see Supplementary Figure S2).

Taken together, our data indicate a potential association between downregulation of the MAPK signaling pathway and low sensitivity of the retina to OIR in FGR neonates.

## Discussion

The main finding of this study was that a relatively low risk of OIR was observed in maternal low-protein-induced FGR neonates. By comparing retinal neovascularization between FGR neonates and neonates from dams fed a normal-protein diet, the susceptibility to pathological vasoproliferation with a disorganized retinal vasculature was shown to be attenuated in FGR neonates. Downregulation of angiogenic factors that trigger pathological retinal neovascularization, such as the MAPK pathway and corresponding upstream signaling pathways, and delayed physiological retinal angiogenesis may be responsible for the low sensitivity of the FGR retina to fluctuating oxygen-exposure. Our results indicated that while facing a struggle between maintaining a safe oxygen saturation and reducing the risk of ROP, an excessive level of supplemental oxygen, outside the current permissible range, could be safe and may be able to be considered in oxygen therapy for premature infants according to their individual needs.

Along with the advancement of neonatal intensive care, the survival rate of premature, particularly extremely premature infants (<28 weeks of gestation), has improved over the years (Reyes et al., 2017). This has directly increased the number of premature infants at risk of developing ROP. Among about 14.9 million live premature births globally, an estimated 184,700 developed ROP, including 20,000 with severe visual impairment or blindness (Blencowe et al., 2013; Brown and Nwanyanwu, 2023). It is essential to balance the benefits of oxygen therapy with the risks of hypoxemia and hyperoxemia (Jobe and Kallapur, 2010). Hypoxemia can cause brain damage, organ failure and even be fatal, while hyperoxemia can damage the retina and other organs. It can be challenging to achieve the optimal level of supplemental oxygen to prevent ROP while maintaining safe oxygen levels in the intensive care of FGR infants.

Supplemental oxygen triggers ROP in low-birth weight preterm infants through two distinct phases. In the first phase, after birth, premature infants lose placental and maternal growth factors. Under a relative hypoxic condition, a network of astrocytes that spreads outward from the optic nerve head before physiological retinal vasculature development provides well-orchestrated gradients of angiogenic factors, including vascular endothelial growth factor (VEGF), a central player in neovascular disorders, insulin-like growth factor-1 (HIF-1), which has been reported to be critically associated with the severity of clinical ROP (Cavallaro et al., 2014), and erythropoietin (EPO), which is a pleiotropic growth factor that confers growth stimulation and cytoprotection to endothelial cells (Romagnoli et al., 2011). Vaso-obliteration is initiated at birth and subsides on termination of supplemental oxygen treatment at approximately 32–34 weeks of gestational age (Dai et al., 2021). After birth, along with exposure to a high environmental oxygen tension, downregulation of the expression of angiogenic factors that support physiological angiogenesis occurs, which thus promotes vaso-obliteration.

Normal neonatal mice are born with a hyaloid retinal vasculature, which is different from the human retinal vasculature. In humans, the retinal vasculature begins to develop at around 16 weeks of gestation and is fully mature within 40 weeks, normally before birth. In murine neonates, the retinal vasculature develops postnatally. The retinal arteries sprout out of the optic nerve head into the retina on P1, followed by outward branching and extension toward the peripheral retina (Sapieha, 2012). The superficial retinal vessels reach the edge of the retina by P8, penetrate into the retina from P7-P12, and subsequently mature into established superficial, intermediate and deep plexuses (Dai et al., 2021). Retinal vasculature development is delayed in FGR neonates under a normoxic condition, as indicated by our observation on P17, and therefore vaso-obliteration due to deficiency of angiogenic growth factors does not appear to be as severe as that in normal controls.

A vaso-proliferation phase, the second phase, subsequently begins at the time of withdrawal of supplemental oxygen (Dai et al., 2021). In a normal condition, astrocytes under hypoxic conditions produce well-orchestrated gradients of angiogenic factors, such as VEGF, HIF-1, and EPO. These factors support physiological angiogenesis in a stepwise manner directed toward the periphery, to form a highly structured retinal vascular network in mice (Sapieha, 2012). In the development of OIR, the burst of angiogenic factors during this phase lacks such regulated temporal and spatial gradients, thereby provoking retinal neovasculature which originates from an immature astrocyte network, resulting in disorganized retinal vasculature. Compared with those in mature neonatal mice with normal-birth weight, impaired protein synthesis and dysfunction of related intracellular signaling pathways due to their immature organ systems and limited nutrient stores in FGR neonates lead to insufficient hypoxia-induced metabolic activation of angiogenic factors (Schmidt et al., 2019). Protein restriction throughout gestation induces a significant reduction in fetal and placental weight, along with suppression of fetal organ development in a murine model, which is relevant to full-term infants with low-birth weight (Gonzalez et al., 2016; Serpente et al., 2021). We previously reported that FGR increases the susceptibility of the vasculature to postnatal injury, with an increase in HIF-1α but a decrease in angiogenesis (Chisaka et al., 2016). Significant downregulation of receptors of growth factors, such as the ErbB family of receptor tyrosine kinases (*RTKs*) and other G protein-coupled receptors which couple binding of extracellular growth factor ligands (Wang, 2017), as shown in the PI3K-AKT signaling map, was observed in FGR neonatal mice on P17 compared to those in normal neonates. In humans, the PI3K/AKT/MAPK pathway is located both upstream and downstream of VEGF signaling and is able to regulate the production and function of VEGF in angiogenic processes (Wang et al., 2014; Zhao et al., 2020), which may consequently delay neovasculature development in FGR neonatal models. The divergence between pathological angiogenesis and the immature astrocyte network is therefore coincidentally compensatory in FGR neonates. This may explain why FGR neonates exhibit tolerance to fluctuating oxygen-exposure. Clinically, concordant with our conclusion, the results of a cohort study showed that the incidence of ROP did not increase in infants with birth weights less than both 1,500 and 1,000 g compared to control (Lin et al., 2022). Our findings indicate that FGR neonates demonstrate a heightened tolerance to fluctuating oxygen exposure when compared to their mature counterparts. Consequently, when confronted with the challenge of balancing the maintenance of optimal oxygen saturation level and minimizing the risk of ROP, stringent restrictions on supplemental oxygen concentration may not be imperative for FGR infants. The results obtained in this study are inconsistent with those reported in other studies conducted in humans, which established that prematurity and low birth weight are major risk factors for the development of OIR (Painter et al., 2015; Trzcionkowska et al., 2021). It is plausible that oxygen concentrations beyond the currently permissible range could be deemed safe and may merit consideration in oxygen therapy for premature infants, tailored to their individual requirements.

One of the major limitations of the present study is that we are not able to validate the massive signaling cascades using other quantification methods, which relate to the mechanisms raised by pathway analysis underlying oxygen-modulated physiological changes in FGR neonates. Also, despite our focus on the single most important risk factor, oxygen, for ROP in the present study, other reported factors, such pulmonary dysfunction, anemia, intraventricular bleeding, and necrotizing enterocolitis, could indirectly influence the onset of ROP (Eckert et al., 2012; Binenbaum, 2013). The overall risk of OIR in premature neonates might thus be underestimated. A comprehensive proteomics approach and systemic evaluation in premature infants are planned for our future studies.

In conclusion, our findings demonstrated that FGR neonates are equipped with higher tolerance to retinal oxygen stress, and the risk of OIR development is attenuated compared to that in mature neonates with normal birth weight. The appropriate balance of neonatal intensive care provisions for oxygen supplementation so that this necessary support for systemic functions does not produce adverse effects such as ROP should be evaluated. With close monitoring and evaluation, individualized optimized supplemental oxygen treatment that favors both a higher rate of survival and proper retinal vascularization in FGR neonates may be achieved.

## Data Availability

The datasets presented in this study can be found in online repositories. The names of the repository/repositories and accession number(s) can be found below: Sequence Read Archive of NCBI. The accession number is PRJNA1007420 (SRR25752107 – SRR25752118).

## Supplementary material

SUPPLEMENTARY FIGURE S1 Representative images of flat-mounted retina in neonates with maternal normal-protein diet (NP) or low-protein diet (LP) exposed to normoxia on P4 (left) and P7 (middle), and hyperoxia on P12 (right). The vasculature was labeled with isolecin B4 (green).

SUPPLEMENTARY FIGURE S2 Immunoblot assay of retinal extracts from NP_75% O_2_ and LP_75% O_2_ neonates. Expression of phosphor-Akt (MW: 60 kD) **(A,B)**, phosphor-PLCγ1 8MW: 116 kD) **(C,D)**, phosphor-p44/42 MAPK (MW: 44/42 kD) **(E,F)**, phosphor-Src (MW: 60 kD) **(G,H)**, HIF-1α (MW: 120 kD) **(I,J)**, and GFAP (MW: 50 kD) was analyzed using a Jess automated simple western blotting system (n = 3). All results are presented as mean ± SD.
