# Supplementary material for: Amelioration of oxygen-induced retinopathy in neonatal mice with fetal growth restriction
Source: Front Cell Dev Biol. 2024 Feb 16;12:1288212. doi: 10.3389/fcell.2024.1288212 (PMC10904624; doi:10.3389/fcell.2024.1288212)
Supplement: Supplementary file 1 [file Table1.DOCX]

|  | **NP** | **LP** |
| --- | --- | --- |
| **Protein (g/100g diet)** | 23  54.7  4.9 | 8.2  73.5  5.8 |
| **Carbohydrate (g/100g diet)** |  |  |
| **Fat (g/100g diet)** |  |  |
| **H_2_O (g/100g diet)** | 8.1 | 8.0 |
| **Others (g/100g diet)** | 9.3 | 4.5 |
| **Total calories (kcal/100g diet)** | 356 | 379 |

**Supplementary Table 1 Compositions of diets used in the study**

NP, normal protein diet; LP, low protein diet
