# Supplementary figures and images for: Amelioration of oxygen-induced retinopathy in neonatal mice with fetal growth restriction
Source: Front Cell Dev Biol. 2024 Feb 16;12:1288212. doi: 10.3389/fcell.2024.1288212 (PMC10904624; doi:10.3389/fcell.2024.1288212)

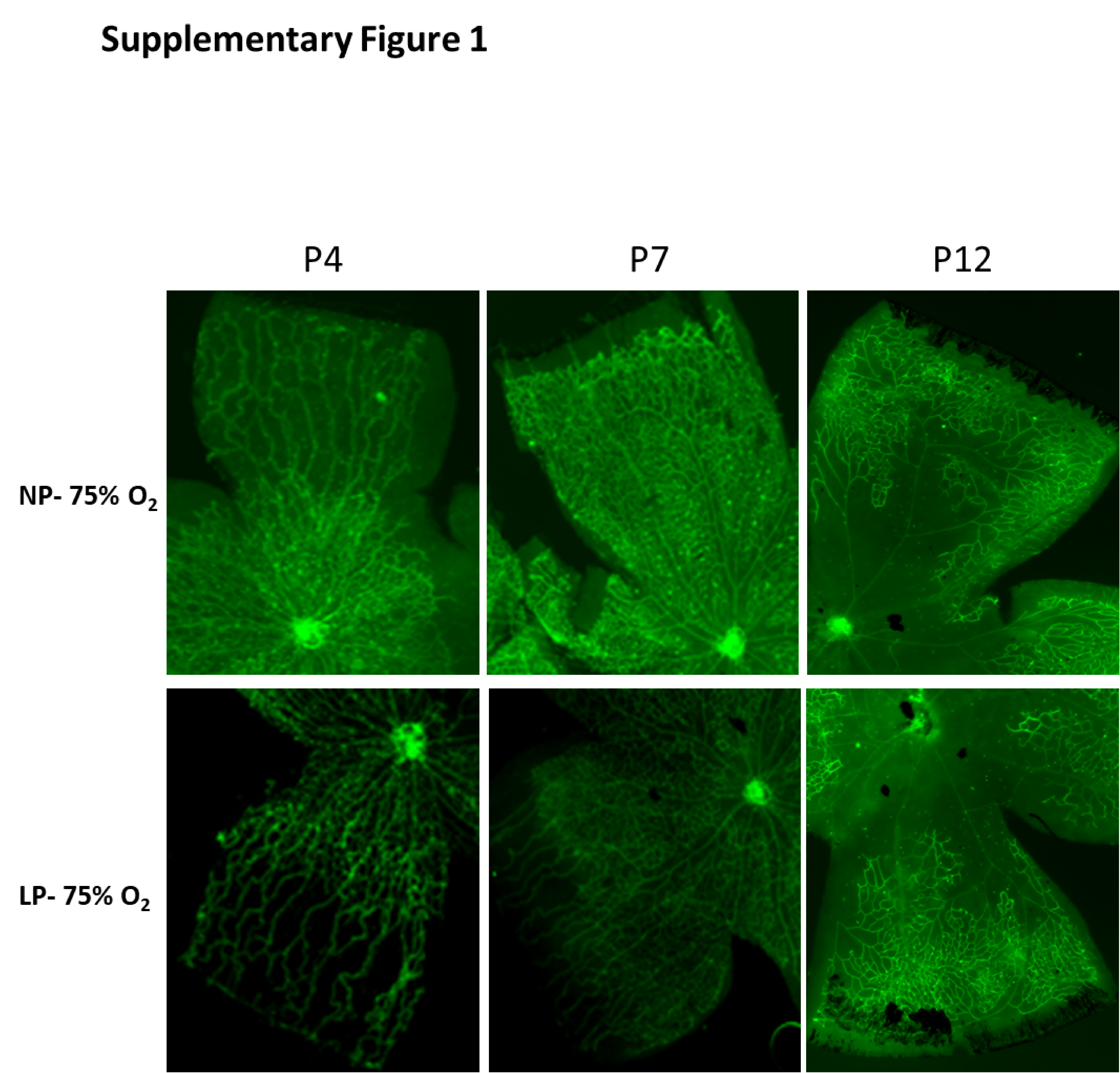

Supplement: Supplementary file 3 [file Image1.TIF]
